# PKCε and allopregnanolone: functional cross-talk at the GABA_A_ receptor level

**DOI:** 10.3389/fncel.2015.00083

**Published:** 2015-03-19

**Authors:** Giulia Puia, Federica Ravazzini, Luca Franco Castelnovo, Valerio Magnaghi

**Affiliations:** ^1^Department of Life Science, University of Modena and Reggio EmiliaModena, Italy; ^2^Department of Pharmacological and Biomolecular Sciences, University of MilanMilan, Italy

**Keywords:** GABAA receptor, phosphorylation site, neurosteroids, receptor traffiking, PKCepsilon

## Abstract

Changes in GABAergic inhibition occur during physiological processes, during response to drugs and in various pathologies. These changes can be achieved through direct allosteric modifications at the γ-amino butyric acid (GABA) type A (GABA_A_) receptor protein level, or by altering the synthesis, trafficking and stability of the receptor. Neurosteroids (NSs) and protein kinase C (PKC) are potent modulators of GABA_A_ receptors and their effects are presumably intermingled, even though evidence for this hypothesis is only partially explored. However, several PKC isoforms are able to phosphorylate the GABA_A_ receptor, producing different functional effects. We focused on the ε isoform, that has been correlated to the sensitivity of the GABA_A_ receptor to allosteric modulators and whose expression may be regulated in peripheral sensory neurons by NSs. The cross-talk between PKC-ε and NSs, leading to changes in GABA_A_ receptor functionality, is considered and discussed in this perspective.

## Introduction

GABA_A_ receptors are fundamental for fast synaptic inhibition in the brain. Given the role of these receptors in synaptic transmission, all the mechanisms that regulate their activity are of primary importance. One that is particularly significant is receptor phosphorylation. Kinases represent a superfamily of isoenzymes that, through protein phosphorylation, regulate several cellular processes, including proliferation, differentiation, tumorigenesis, cytoskeletal remodeling, receptor function and synaptic transmission modulation (Battaini, 2001). This superfamily comprises the protein kinase (PK) A, G and C, which are serine/threonine phosphotransferases.

Protein kinase C (PKC) is one of the most significant kinases for GABA_A_ receptor modulation. Phosphorylation of the receptor can produce different effects, ranging from enhancement to inhibition of protein function, depending on the subtype of subunit targeted and on the location of the sites being phosphorylated (Moss and Smart, 1996).

In particular, among PKC enzymes there are conventional (α, βI, βII and Υ) and new (δ, ε, η and θ) isoforms (Nishizuka, 1995), that are differently expressed in the nervous system. The differences among PKC isoforms contribute to the large range of functions they may perform.

Some PKCs directly bind to the intracellular domain of specific GABA_A_ receptor subunits, providing in this way a rapid regulation of the receptor activity by all the intracellular pathways that activate this kinase (Brandon et al., 1999).

## PKC-Epsilon (PKC-ε)

PKC-ε is classified as a novel isoform present in the brain, found mainly in the cerebral cortex, cerebellum, hippocampus and, in small amount, in non-nervous peripheral tissues (Saito et al., 1993; Chen et al., 2000).

The characterization of PKC-ε null mice has provided insights into the role of PKC-ε in the central nervous system (CNS; Hodge et al., 1999). Animals lacking PKC-ε show hypersensitivity to the behavioral effects of allosteric modulators of GABA_A_ receptors, such as ethanol and neurosteroids (NSs; Hodge et al., 2002). Among NSs, the progesterone metabolite Allopregnanolone (Allo) is one of the most important endogenous steroid in the CNS (Baulieu and Robel, 1990), as changes in its concentration correlate with physiological and pathological conditions(Maguire and Mody, 2007; Luchetti et al., 2011).

**Figure 1:**
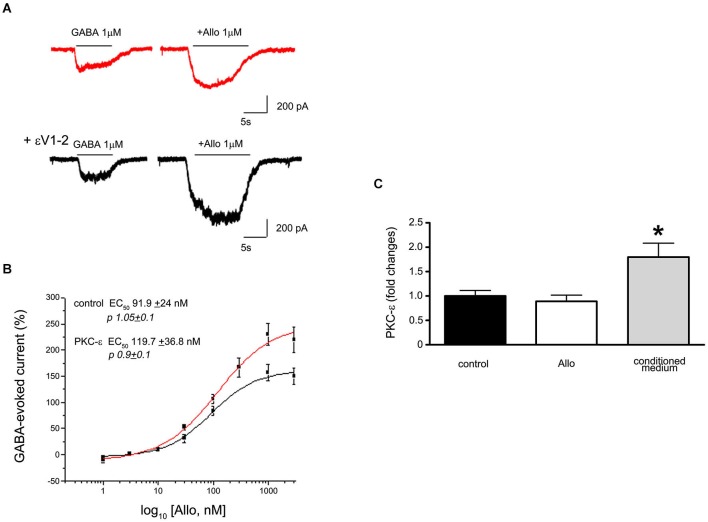
**(A)** Electrophysiological traces showing the effect of Allo on GABA-evoked currents recorded from cortical neurons in culture (7DIV). The upper trace shows a recording from a control cell (red) and the lower trace is from a cell intracellularly perfused with the peptide εV1–2, a PKC-ε inhibitor. **(B)** Dose response curves of Allo effect in control and in the presence of the peptide eV1–2. Each point is the mean+/−SE of 8–15 cells. PKC-ε blockade did not change the potency of Allo but affected its efficacy. **(C)** Assessment of PKC-ε gene expression changes (as fold changes) in primary culture of dorsal root ganglia (DRG) neuron after 24 h exposure to 10^−6^ M Allo (white column), or to the culture conditioned medium obtained from Schwann cell cultures treated with 10^−6^ M Allo (gray column). The relative quantification of mRNA was obtained by quantitative real time PCR. Data were normalized to the housekeeping genes α-tubulin and β2-microglobulin and expressed as difference (^ΔΔ^Ct) vs. controls, then averaged for each experimental group. The black column represents the controls (DRG neurons treated with vehicle, ethanol). Experiments were repeated at least three times (*p < 0.05 Anova Test).

*In vitro* studies on cortical synaptosomes demonstrate that a peptide able to inhibit PKC-ε translocation (Khasar et al., 1999) produces an increase in Allo sensitivity (Hodge et al., 1999). Similarly, in primary cultures of cortical neurons Allo is more effective in potentiating GABA-evoked current when the cells are intracellularly perfused with this same inhibitory peptide (Figure 1A). From analysis of the dose-response curves of this Allo effect, it is evident that the potency of NSs remains unchanged after blocking PKC-ε translocation, whereas the efficacy is increased (Figure 1B). We cannot determine whether the receptors mediating the increased response to Allo were at synaptic or extrasynaptic sites. However, it is possible that a selective phosphorylation by PKC-ε occurs, in this case the kinase activity determines selective changes only in synaptic or in tonic currents. The use of a heterologous system, expressing different GABA_A_ receptor subunits, or specific agonists for “tonic receptors” could provide the answer to this issue.

Recent studies showed that PKC-ε action depends on the phosphorylation of the GABA_A_ receptor at the level of Ser327 of the γ2 subunit (Qi et al., 2007), in turn regulating the response of the receptors to allosteric modulators. Furthermore, PKC-ε kinase controls GABA_A_ receptor trafficking through the N-Ethylmaleimide-Sensitive Factor (NESF)-signaling pathway. Indeed, as suggested by the changes in Allo efficacy from *in vitro* studies (Figure 1B), the activation of PKC-ε is able to decrease cell surface expression of these receptors (Chou et al., 2010).

## NSs and Phosphorylation

Allo, and other NSs that are positive modulators of GABA_A_ receptors, potentiate GABA-evoked chloride current through an increase in the channel opening probability (Puia et al., 1990; Twyman and Macdonald, 1992; Zhu and Vicini, 1997). This results in a prolongation in the decay time of inhibitory post-synaptic currents (IPSCs; Harrison et al., 1987; Fáncsik et al., 2000). However, NSs also act on extrasynaptic GABA_A_ receptors, causing large effects on δ-containing receptors that mediate the tonic current in certain brain regions (Belelli et al., 2002; Stell et al., 2003).

Interestingly, the desensitization of GABA_A_ receptors plays an important role in NSs’ modulation (Zhu and Vicini, 1997), suggesting that the receptor needs to be in a “specific” state to be responsive to these endogenous NSs.

Furthermore, NSs produce long-lasting changes in the efficacy of GABAergic neurotransmission by modulating the phosphorylation of synaptic and extrasynaptic receptors. Indeed, in a recent paper, Abramian et al. showed a new molecular mechanism by which NSs change the efficacy of GABAergic inhibition by increasing surface expression of specific GABA_A_ receptors (mainly containing α4 subunits), responsible for the tonic current in hippocampus (Abramian et al., 2014). They suggested that this component of GABAergic neurotransmission may be a key regulator of excitability. In this way the phosphorylation process may alter the function and/or trafficking of GABA_A_ receptors, thus changing the efficacy of GABA_A_-mediated inhibition.

Interestingly other allosteric modulators of GABA_A_ receptors, i.e., benzodiazepine, may also change GABA signaling, influencing the diffusion and clustering of receptors at synapses (Lévi et al., 2015). Conversely, it was shown that the potentiating effect of NSs can be decreased after stimulation of the PKC signaling pathway, either in physiological (Brussaard et al., 2000; Brussaard and Koksma, 2003; Maguire et al., 2005; Oberlander et al., 2012) or pathological conditions (Mtchedlishvili et al., 2001; Kia et al., 2011). For example, the phosphorylation state of the GABA_A_ receptor changes during pregnancy or over the estrous cycle, a phenomenon that can compromise GABA_A_ modulation by NS action.

The modulatory efficacy of NSs is decreased in dentate granule cells from epileptic rats (Mtchedlishvili et al., 2001). This agrees with the observation that brain and cell specific changes in GABA_A_ receptors may occur in several epileptic models (Schwarzer et al., 1997; Fritschy et al., 1999; Peng et al., 2004) and in the temporal lobe of human epileptic subjects (Loup et al., 2000; Ferando and Mody, 2012). Recent studies show changes in the phosphorylation of GABA_A_ receptors after kindling; these modifications together with changes in GABA_A_ subunit composition could account for a decreased responsiveness to NSs (Kia et al., 2011; Carver et al., 2014). The diminished sensitivity to endogenous positive allosteric modulators, such as Allo, increases susceptibility to seizures, similarly to what happens in women with catamenial epilepsy, where seizures occur more frequently before the onset of menses and NS levels fall due to progesterone crash (Reddy, 2009).

Also important is the cross-talk among PKC-ε, GABA_A_ receptors and NSs in pain perception. NS production is stimulated as a result of inflammatory pain (Poisbeau et al., 2005), and accordingly changes in PKC-ε expression were observed during pathological pain (Parada et al., 2003). Vergnano et al. proposed that the 3α-5α-NSs (such as Allo) could be part of an endogenous compensatory mechanism, in response to sustained activation of the spinal nociceptive system that occurs during pathological conditions (Vergnano et al., 2007).

The persistence of incoming pain messages, and the associated increases in intracellular Ca^2+^ concentration, could induce a strong stimulation of Ca^2+^-dependent PKC. This leads to a functional block of GABA_A_ receptors in their current state. Therefore, PKC prevents further 3α-5α-NS dependent potentiation, without decreasing the basal modulatory effect of 3α-5α-NSs (Vergnano et al., 2007).

PKC-ε exerts a modulatory action on the pain pathways acting on CNS neurons, but also at the peripheral nervous system (PNS) level, for instance on dorsal root ganglia (DRG) neurons. Indeed, PKC-ε may alter the permeability of Na-type Ca^2+^ channels in DRG, and it has been shown to enhance nociception (Van Kolen et al., 2008). The capability of endogenous mediators to regulate PKC-ε gene expression has already been demonstrated for several molecules, including the neuropeptide ghrelin, thyroid hormones, the apolipoprotein E3 and some miRNAs (Rybin and Steinberg, 1996; Alipour et al., 2011; Sen et al., 2012). However, no data reported on the possible modulation exerted by NSs on PKC-ε gene expression. Recent studies by qRT-PCR analysis evaluated the possible modulation of PKC-ε expression in DRG neuronal cultures following 24-h treatment with Allo (10^−6^ M). Allo did not change PKC-ε expression under basal conditions, but was significantly up­regulated in DRG neurons exposed to the culture medium from Allo-treated Schwann cells (Figure 1C). These findings suggest that Allo-treated Schwann cells can release one or more factors able to modulate PKC-ε expression in DRG neurons. However, Schwann cells also express basal levels of PKC-ε (Borghini et al., 1994). Overall, we speculate that these mechanisms identify novel putative circuits involved in the regulation of pain processes at PNS and spinal cord levels.

Given that Allo is considered one key factor in the modulation of peripheral pain pathways, its capability to regulate PKC-ε is promising and opens new perspectives for the identification of the basic mechanisms regulating chronic pain onset.

## Conclusions

The activity of GABA_A_ receptors must be finely regulated in the CNS. For this reason when NSs increase GABA_A_ receptor activity, the “system” tries to re-equilibrate by activating different PKs. The increase in neuronal activity that occurs by activating L-type Ca^2+^ channels leads to a Ca^2+^/calmodulin-dependent PK type II phosphorylation of the GABA_A_ receptor β3 subunit. This in turn produces a rapid insertion of receptors in the membrane, with a consequent increase in tonic current (Saliba et al., 2012). Similarly, NSs promote GABA_A_ receptor phosphorylation, leading to an increase in extrasynaptic receptor expression (Abramian et al., 2014).

These findings shed light on a new type of modulatory activity played by NSs. Indeed, NSs not only allosterically modulate GABA_A_ receptor function and synthesis, but can also regulate membrane trafficking of the receptor protein, which is particularly important in determining synapse efficacy. Another NS, pregnenolone sulphate, uses the same “strategy” to modulate N-Methyl-D-aspartate (NMDA) receptor-mediated neurotransmission. Indeed, its effects are determined by direct modulation of the NMDA receptor, but also by increasing receptor expression on the cell surface (Kostakis et al., 2013).

Interestingly, in physio-pathological situations, or after pharmacological treatments, NSs (endogenous and exogenous) and PKC activities may vary a lot. The precise role of functional cross-talk between these “modulators” (Adams et al., 2015) and how these interactions can affect GABA_A_ receptor function are still a matter of investigation.

**Figure 2:**
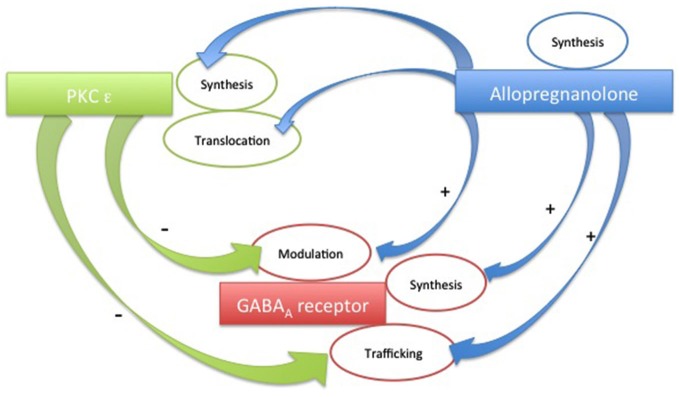
**Schematic plot of the possible cross-talks among PKC-ε, Allopregnanolone and GABA_A_ receptors**.

In Figure 2 we summarize some of the interrelationships among these three players. We believe that it is important to keep in mind these pathways. Eventually, shedding light on other presently unknown cross-talks will help to better understand the mechanisms underlying some neuropathologies, as well as unraveling the mechanisms of action of novel GABA_A_ modulating drugs. However, a complex picture emerges from the recent findings. The pharmacological response to endogenous molecules or to exogenous drugs results from the dynamic interrelationship between modulators and receptor proteins. This cross-talk can produce different responses, depending on factors such as cellular distribution or subtype and phosphorylation state of the receptor involved.

As a general perspective, the importance of GABA_A_ receptor phosphorylation is particularly relevant when a pharmacological treatment involving allosteric GABA_A_ receptor modulators, such as NSs, is started. However, it should be emphasized that GABA_A_ receptor rearrangement at the synapse level, and/or changes in receptor subunit composition, can lead to different pharmacological effects. Altogether, these hypotheses should be taken into account to better understand the complex behavior of NSs at the level of neuronal circuitries (Puia et al., 2012) and in *in vivo* studies.

## Conflict of Interest Statement

The authors declare that the research was conducted in the absence of any commercial or financial relationships that could be construed as a potential conflict of interest.
